# Case Study of Tobacco Use among Myanmar Migrant Factory Workers in the Seafood Industry in Thailand

**DOI:** 10.3390/ijerph18168659

**Published:** 2021-08-16

**Authors:** Naowarut Charoenca, Nan Khin Thet Chaw, Nipapun Kungskulniti, Stephen L. Hamann

**Affiliations:** 1 Faculty of Public Health, Mahidol University, Bangkok 10400, Thailand; naowarut.cha@mahidol.ac.th; 2 Center of Excellence on Environmental Health and Toxicology, Bangkok 10400, Thailand; 3 International Rescue Committee (IRC), Mae Hong Son 58000, Thailand; nanglaotai1923@gmail.com; 4 Tobacco Control Research and Knowledge Management Center, Bangkok 10400, Thailand; slhamann@gmail.com

**Keywords:** smoking, Myanmar, migrants, psychosocial factors, non-communicable disease burden

## Abstract

Migrant workers commonly face many health disparities when they relocate to a foreign work environment. Many workers migrating to Thailand are young unskilled workers from Myanmar. In this study, we examine factors associated with Myanmar migrant workers’ smoking status and characterized smoking-related knowledge, attitudes, and behavior in one seafood factory in Thailand. This descriptive study utilized person-to-person interviews among 300 Myanmar migrants in one seafood factory in Thailand, of which 94.3% were young males between 18 and 39 years of age. Results demonstrated that 90% were current daily smokers, over 90% smoked 30–60 times per month, and 95% spent less than 500 baht (US $16) per month on smoking. About 70% of current smokers had 6–10 friends who smoked, compared with 40% of non-smokers (chi-square, *p*-value ≤ 0.07). Among this sample of mainly male migrant workers, smoking is very common, in part driven through social contact, but levels of dependence appear relatively low. The results suggest potential intervention approaches to reduce high smoking prevalence among this population, such as targeting young males and addressing their concerns about negative attitudes by peers to tobacco use and the unhealthful exposures of women and children in their families and the larger community.

## 1. Introduction

Tobacco-related illness is the leading cause of death of more than eight million people worldwide each year [1]. About 80% of the world’s 1.3 billion smokers are from low- and middle-income countries (LMICs), with most from Asia [2]. Nearly one-fifth of smokers worldwide are from the Southeast Asia region [3]. Non-communicable diseases (NCDs) are rapidly increasing in LMICs, and smoking plays a major role in all major NCDs (cardiovascular disease, cancer, respiratory disease, and diabetes) [4].

Migrant populations are increasing worldwide, including in Thailand. In 2019, there were 277 million migrants, with 60% born in Asia and most relocating to countries in Asia [5]. In 2019, Thailand had an estimated 3.9 million documented and undocumented migrant workers, mostly from Cambodia, the Lao People’s Democratic Republic, Myanmar, and Vietnam [6]. According to Thailand’s labor ministry, there were a total of 2.5 million migrant workers registered in the kingdom at the end of 2020. Of those, more than one million were from Myanmar, making Thailand host to the largest Burmese expatriate community in the world [7]. Data from Samut Sakhon Province where most seafood factories are located in Thailand indicates that there are 225,600 Myanmar migrant workers in the province. The seafood factory in our study had 1619 (79%) Myanmar migrant workers of a total factory workforce of 2050 workers [8].

Research shows the devastating effects of tobacco use and secondhand smoke exposure on the body [9]. Many tobacco users are unaware of the harmful chemicals in tobacco products or their negative health consequences [10,11]. Lacking awareness of tobacco use dangers, many migrants use smoking as a coping mechanism to the stress of moving and a new workplace [12]. While sometimes smoking rates may increase among migrants due to stressful work conditions or acculturalization, in many cases, migrants’ health behaviors may improve as they adopt healthier lifestyles prevalent in the new country [12,13]. For example, if male migrants adopted the male smoking norms of Thailand, rates of smoking should drop from 79.6% for males in Myanmar to 38.6%, the rate for males in Thailand [3].

Migrant populations often lack the same level of access to healthcare services as the native population. Numerous acute and chronic conditions may occur among migrants. Studies of migrants in Thailand have demonstrated how tuberculosis, reproductive health, and mental health factors disproportionately affect Myanmar migrant populations [14,15,16,17]. International health authorities have noted the need for increased attention to migrant health. For example, the World Health Organization (WHO) strongly recommends “that health checks be offered and provided to ensure access to health care for all refugees and migrants requiring health protection. Checks should be performed for both communicable diseases and NCDs, while respecting the human rights and dignity of refugees and migrants” [18]. The lack of health services was related to the recent outbreak of COVID in Thailand among seafood industry workers from Myanmar and caused additional hardships for them [7,19].

Thailand benefits significantly from migrants who “help fill labor shortages, contribute to economic growth and are becoming ever more important as Thai society ages. Constituting over 10 per cent of the total labor force, their work is thought to contribute between 4.3 to 6.6 per cent of Thailand’s Gross Domestic Product” [6]. Since reduced smoking rates produce both personal benefits for Myanmar migrant workers and also Thailand’s economic success, understanding the factors that affect smoking is useful in similar contexts worldwide [20]. This study aims to characterize smoking status and the factors that influence smoking-related behaviors among a sample of male migrant workers from Myanmar working in the seafood industry in Thailand. This case study provides a good opportunity to understand the segregated migrant work circumstances that may influence tobacco use behaviors.

## 2. Materials and Methods

This descriptive study was conducted in Samut Sakhon Province, where Myanmar migrants make up the largest group of foreign laborers. Due to its location in the Gulf of Thailand, there are many seafood factories in the city. One large seafood factory with 2050 employees agreed to let employees from Myanmar participate in the study. This purposive sample reflects a common migrant population in Thailand. A sample size of 300 subjects of the 1619 Myanmar migrant workers at the factory was chosen using a formula from Daniel (1999) for calculating the sample size [21], based on smoking prevalence among Myanmar migrant workers in Mahachai, Thailand [22]. Inclusion criteria are as follows: born in Myanmar, living in Samut Sakhon for at least six months, aged 18–55 years, speaking the Burmese language for communication, and working at the factory during the day.

Following IRB approval (COA No. MUPH 2015-091) by the Ethics Committee of the Faculty of Public Health, Mahidol University, the factory allowed the research team to conduct person-to-person interviews using a structured questionnaire on smoking status, knowledge, and behavior. The questionnaire was developed by the researchers, and reviewed by independent public health experts. The questionnaire’s validity was assessed by public health experts, and the reliability was confirmed by a pretest using Cronbach’s Alpha Coefficient test (0.68). Then, the questionnaire was translated into Burmese and back-translated by Myanmar public health colleagues with corrections as necessary. The researcher who conducted the interviews was from Myanmar.

The questionnaire consisted of 64 questions in four major areas: (1) socio-demographic characteristics, (2) smoking status, (3) personal knowledge and attitudes, and (4) psychological factors. Questions were designed to provide categorical (frequency and percentage) and continuous responses (mean and standard deviation).

Socio-demographic factors included age, gender, marital status, education, type of employment position, income as a day worker, and duration of stay in Thailand (seven questions).

Smoking was defined as using manufactured or hand-rolled cigarettes or smokeless tobacco, which was less commonly used. Smoking status is classified into three groups: current smokers, ex-smokers, and non-smokers. Current smokers were those currently smoking or those who had smoked in the 30 days before data collection. Ex-smokers were those who had smoked previously but did not smoke at the time of the interview and had not smoked in the last month or longer. Non-smokers were those who did not currently smoke and who had never used any tobacco products. Smoking status included current smoking status, age of initiation, type of tobacco product used, length of time smoking, days of smoking per week, frequency of smoking in the last month, time of smoking after waking, most common time of day to smoke, whether smoking even if sick, smoking in relation to friends and family, reasons for smoking or not smoking (non-smokers), and money spent on smoking each month (27 questions).

Personal factors included knowledge of the harms of tobacco use in 10 specific areas and attitudes toward tobacco consumption in 10 specific areas (20 questions).

Psychological factors included questions on stress as with problems sleeping, concentrating, anxiety, boredom, and social interactions. In addition, questions on overwork, peer pressure to smoke, and family influences on smoking were included (10 questions). Personal and psychological factors in the questionnaire were derived from standardized survey instruments from the USCDC, the IARC Tobacco Policy Evaluation Handbook, and the DASS 21 Handbook. Questions for smoking status were from the US CDC Global Adult Tobacco Survey questions. Fagerstrom Dependency questions were used to assess dependence level, and DASS-21 questions were used to assess anxiety and stress. We used SPSS to calculate chi-square results showing the association of categorical variables [23,24,25,26,27].

## 3. Results

### 3.1. Socio-Demographic, Smoking, and Workplace Factors

Of the 300 participants, 90% were current smokers, with a mean age of 27 ± 5 years of age. Of the 270 current smokers, 98.5% were male. Most respondents were married (73.3%), with 80.7% having only secondary education or below. Both male gender and secondary education or less were statistically significantly related to tobacco use. All subjects were daily factory workers who made between 200 and 300 baht per day (US $6–10). These migrant workers had resided in Thailand for an average of fewer than 10 years (mean = 6.4 ± 3.4).

Smokers’ mean age of initiation of tobacco use was 18.59 years (SD ± 2.08). Most smokers (77.8%) reported smoking for less than 10 years, while 22.2% reported smoking more than 10 years; most were daily smokers (90.7%) with only 5.6% using tobacco products 4–6 days per week, and 3.7% using them 1–3 days per week.

Most current smokers (66.7%) smoked 30–60 times per month. Most smoked after meals during the day (73.4%), while 26.6% smoked when meeting with friends and family members in the evening after work. Only one-third of smokers never smoked near family members at home or in public places or social events. Between 62.6% and 75.6% of smokers smoked in social settings such as weddings and parties. One exception to social smoking was that most (94.0%) said they did not smoke in the presence of children. Most smokers stated that they did not encourage others, family members, or friends to smoke (90.3%). Smokers claimed that they spent less than 500 baht (US $16) per month on smoking (94.0%). This is less than 7% of their monthly wage and even less than half that if they use roll-your-own tobacco. In terms of reasons for using tobacco products, most (70.4%) stated that they smoked for taste or pleasure; most of the rest indicated that they smoked because they craved or were addicted to tobacco.

Table 1 shows that the three types of tobacco products were most commonly consumed by smokers, which were manufactured cigarettes (31.5%), hand-rolled cigarettes (30%), and smokeless tobacco (27%), while a small percentage were dual users. The majority of smokers (51.5%) indicated that their first cigarette or tobacco product use was in the morning, most often more than 30 min after waking up but before noon. However, most smokers (94.1%) did not use cigarettes or tobacco products when feeling sick.

The results of knowledge about tobacco use in Table 2 show that 80% or more of migrant tobacco users had general knowledge about tobacco use harms in six areas but had more limited knowledge about the addictiveness of tobacco, risks for diabetes, effects on non-smokers, and tobacco’s contribution to diseases other than lung cancer and heart disease.

The results of attitudes in Table 3 demonstrate that migrant tobacco users had negative attitudes on tobacco use and recognized that it affected health, concentration, social interactions, and dependence. Positive attitudes were expressed for tobacco use as a way to make friends (73.3%), as a relaxant (56.2%), and for relief from worries (48.3%).

### 3.2. Psychosocial Factors and Tobacco Smoking Status

Nearly 100% of the subjects worked 8–10 h per day, and only 10.7% indicated that they experienced high stress. The long working hours and level of stress did not appear to differ by smoking status though there were too few non-smokers to make a statistically meaningful comparison. At this seafood factory, the working hours of migrant workers are 8 h a day, and they receive overtime pay for each hour of overtime worked. Therefore, migrant workers were comfortable with the present workload at the factory, and most did not work at any other places. Smokers had friends who were almost entirely smokers, while only two-thirds of non-smokers’ friends were smokers. Current smokers had many more smoking friends. Nearly 70% had 6–10 smoking friends, while only 40% of non-smokers had 6–10 such friends. Neither smokers nor non-smokers (around 94%) had been encouraged to smoke by their friends. The vast majority of both smokers (86.3%) and non-smokers (95.2%) had only one additional smoker in their family. Nearly all workers (99%), both smokers and non-smokers, did not support the idea of smoking by family members.

## 4. Discussion

The unusually high prevalence of current smokers (90%) was higher than findings in a previous study among adult Myanmar migrant workers in Mahachai sub-district, Samut Sakhon Province, which reported an overall prevalence for both sexes of current smokers of 35.2%, ex-smokers 2.3%, and non-smoker 62.5% [22]. Another study of Myanmar migrants in Northern Thailand showed that only 26.9% smoked [20]. These lower smoking rates may be explained by the lower smoking rate of one ethnic group of Myanmar migrants who worked in Northern Thailand and the fact that many more non-smoking females were included in the population sample of this study. These discrepancies with our results may be explained by differences in ethnic/regional Myanmar origins and that 93% of our participants were male, which have a much higher smoking rate as compared to females. In fact, Myanmar has the highest male smoking rate in Southeast Asia at 79.8% [4]. Another explanation for this difference might be that participants in our study were from the same factory and housing area. Workers gathered during lunch and break times, providing them with a ready social environment for smoking together compared to others from less insulated communities that do not work and live together.

Smoking initiation occurred at a mean age of 18.59 years for 53.7% of migrants, consistent with tobacco initiation in Myanmar at the age of 19 [4]. Most workers began smoking before or early in their work experience in Thailand. This age was similar but slightly younger than what was observed in the Mahachai study (19.3 years old). Older age of initiation might have occurred due to more women smokers surveyed in the Mahachai study. Women from Myanmar generally start smoking later in life, at an average age of initiation of 22.3, the oldest age of initiation in countries of the Southeast Asia region [4].

Most smokers were daily tobacco users (90.7%), with only about 10% occasional tobacco users. These findings were similar to the results obtained in a previous study of Myanmar youth workers in Mae Sot District of Tak Province, Thailand [12]. In our study, 78.8% were daily smokers, with 11.9% using 4–6 times a week and 9.3% using 1–3 times per week. Frequent smoking may be higher because tobacco is readily available and affordable in a local migrant community in Thailand, resulting in smoking for stress relief and leisure.

Since most daily smokers started smoking before age 19, they had smoked less than 10 years, with 30% smoking less than 5 years. They had tried and used different tobacco products, including manufactured cigarettes, hand-rolled tobacco, and smokeless tobacco. Each type was currently used by about 30% of the tobacco users. This usage is different from the Thai population, where smokeless tobacco is little used, and manufactured cigarettes predominate. While tobacco products have become more affordable in Myanmar, Thailand’s taxes on manufactured cigarettes are the highest as a share of total taxes in the retail price of any Southeast Asian country [4]. However, the Thai tax on hand-rolled tobacco is meager, allowing low-wage earners to spend little on tobacco as a percentage of their salaries if they buy and use hand-rolled tobacco [28]. Since most workers had low wages, roll-your-own (loose cigarette) tobacco was more common in these smokers (30%). Smokeless tobacco was used more likely due to this being culturally accepted in Myanmar, as in Nepal, Bhutan, and India [4].

A high percentage of smokers had low or moderate knowledge about tobacco use, compared to non-smokers who had better understanding of tobacco harms. Since most workers were smokers, their tobacco usage likely dissuaded them from learning about tobacco use dangers. However, providing knowledge about tobacco use to Myanmar migrant workers could help reduce their tobacco consumption. This would be consistent with other studies that found a difference in smoking knowledge between smokers and non-smokers. In a study from Taiwan, participants who had a better understanding of smoking had a lower risk of smoking [29].

While migrants viewed tobacco use as a health risk, they also viewed it as facilitating social interactions. Negative attitudes to tobacco use were considerable for health consequences, as shown in Table 3, but there were ambiguous attitudes when it came to tobacco use for personal relief from worry and relaxation, including for social relations with friends. The findings that negative attitudes to tobacco use lead to less use are consistent with this direct relationship of greater knowledge and less use in research even after adjusting for potential confounders [29].

The study findings revealed that peer influences are statistically associated with tobacco smoking status, as seen in Table 4. Those who had friends who smoke were more likely to be smokers than those who did not have friends who smoke. Moreover, those who had 6–10 friends who smoke were more likely to be smokers (69.4%) than those who had only 1–5 friends who smoke (30.6%). In this study, workers claimed that they did not encourage their friends to smoke, and smokers denied any influence from friends to use tobacco.

The Mahachai sub-district study in Samut Sakhon Province [22] showed that 53.5% of subjects with close friends who smoke were current smokers, and of those with no close friends who smoke, only 5.3% were current smokers. In this study, nearly all subjects smoked, so peer influence for initiation could not be determined except by asking about encouraging others to smoke. Workers stated that they did not advocate smoking to their friends and had not tried to persuade them to smoke. There was an extremely high acceptance of tobacco use in most social circumstances (markets, parties, and weddings) in this migrant community. Only smoking in the presence of children was considered unacceptable behavior. This potential harm to children is perhaps one behavior that could be used to educate these workers about tobacco smoke harm and the idea of smoking cessation. Where smoking has been normalized, health promotion campaigns should address both the individual and social reasons for not smoking. Health promotion campaigns should be in the Burmese language and utilize pictures to illustrate information relevant to migrant circumstances and the ability to act on this information.

## 5. Conclusions

Based on early smoking initiation and existing country prevalence, male migrants from Myanmar should be more targeted for smoking prevention and cessation education. Tobacco control programs should be introduced which target all migrants, but males more than females because of cultural acceptance of smoking by males. Those with less than a secondary school education should be provided information on the health and economic consequences of tobacco use and how to quit. In studies including Thai low-wage factory workers, “occupational status is closely related to individual educational level and the association between occupational status and smoking in men … was found to be significant” [30]. Community or workplace education and cessation programs could be provided depending on the migrant community’s availability and stage of readiness. The prevalence of tobacco consumption among young adults aged 18–39 years was higher than in the older age group (40–50 years). Because most smokers are young adults with a mean age of 27, appropriate educational campaigns should be designed with this age group in mind.

Among personal factors, knowledge and attitudes were important factors related to tobacco smoking status. Findings suggest that less than half of the respondents know that smoking can cause other diseases besides lung cancer and heart disease and do not understand the harms of secondhand smoke. Moreover, about two-thirds of respondents believed that people who use tobacco have more friends.

Limitations of this study include finding results of a special population, a strength in exposing the real conditions of this environment, that limited generalizability. The lack of female subjects and never-smokers (subject homogeneity) was unanticipated. The inclusion of measures for testing hypotheses evident from past studies would have been useful in the initial design.

Among mainly male migrant workers, smoking is very common, in part driven through social contact, but levels of dependence were relatively low. The results suggest potential intervention approaches to reduce high smoking prevalence among this population, such as targeting young males and addressing their concern for negative attitudes by peers to tobacco use and the unhealthful exposures of women and children in their families and the larger community.

## Figures and Tables

**Table 1:** Smoking pattern and behavior of smokers.

Smoking Pattern (*n* = 270)	*N*	Percent
Type of tobacco products used currently		
Manufactured cigarettes	85	31.5
Hand-rolled cigarettes	81	30.0
Smokeless tobacco	73	27.0
Manufactured cigarettes and hand-rolled cigarettes	15	5.6
Manufactured cigarettes and smokeless tobacco	12	4.4
Time using cigarettes or tobacco products after waking		
Within five minutes	18	6.7
More than 30 min after waking but before noon	139	51.5
In the afternoon	102	37.8
In the evening	11	4.0
Smoking when feeling sick		
Yes	16	5.9
No	254	94.1

**Table 2:** Number and percentage of subjects with correct knowledge about tobacco use based on the rating of factual statements (*n* = 300).

	Correct Answer	
Statements of Knowledge	*N*	Percent
1. Smoking affects the pregnant woman and her babies	295	98.3
2. Inhaling smoke from other persons smoking is harmful	282	94.0
3. Only adults over 18 should be allowed to purchase tobacco products	271	90.3
4. Smokers have a high risk of developing lung cancer and heart disease	260	86.7
5. Smoking can cause cancer anywhere in our human body	248	82.7
6. Consuming chewable tobacco causes oral cancer	244	81.3
7. Tobacco smoking is really addictive	181	60.3
8. Smoking is a risk for diabetes	116	38.7
9. Smoking only affects smokers	111	37.0
10. Smoking can cause other diseases than lung cancer and heart disease	85	28.3

**Table 3:** Number and percentage of positive/negative attitudes on tobacco use (*n* = 300).

Attitudes on Tobacco Use	*N*	Percent
*Subjects disagreed with these statements on tobacco use*		
Smoking/tobacco use will not affect one’s health	263	87.7
Smoking increases the ability to concentrate	188	62.7
Smoking makes people look more fashionable or attractive	179	59.7
Smoking increases social interactions	167	55.7
Tobacco use is a way for people to express their independence	167	55.7
Smoking makes people look more grown-up	155	51.7
*Subjects agreed with these statements on tobacco use*		
People who use tobacco have more friends	220	73.3
Tobacco smoking is an acceptable way for relaxing	169	56.3
Smoking helps you to forget your worries	145	48.3
Smoking helps one to avoid obesity	106	35.3

**Table 4:** Psychosocial factors and tobacco smoking status.

	Smoking Status				*p*-Value
Psychosocial Factors	Current Smoker (*N* = 270)		Non-Smoker (*N* = 30)		
	Number	Percent	Number	Percent	
Average hours of working per day					0.900
8–10 h	269	99.6	30	100.0	
More than 10 h	1	0.4	0	0.0	
Perceived level of stress					0.056
Low and moderate	238	88.1	30	100.0	
High	32	11.9	0	0.0	
Peer pressure					
Smoker friends					<0.001
Yes	255	94.4	20	66.7	
No	15	5.6	10	33.3	
Smoker friends among 10 closest friends	*n* = 255 *		*n* = 20 *		
1–5	78	30.6	12	60.0	0.070
6–10	177	69.4	8	40.0	
Friend persuade to smoke					0.588
Yes	17	6.3	2	6.7	
No	253	93.7	28	93.3	
Family influence					
Smokers in the family					0.218
One	132	86.3	20	95.2	
Two or more	21	13.7	1	4.8	

255 * and 20 * are from the “yes” smoker friends category above.

## Data Availability

The data is available through the authors.
